# A document processing pipeline for annotating chemical entities in scientific documents

**DOI:** 10.1186/1758-2946-7-S1-S7

**Published:** 2015-01-19

**Authors:** David Campos, Sérgio Matos, José L Oliveira

**Affiliations:** 1 BMD Software, Lda., Rua Calouste Gulbenkian, 1, 3810-074 Aveiro, Portugal; 2 DETI/IEETA, Universidade de Aveiro, Campus Universit´ario de Santiago, 3810-193 Aveiro, Portugal

**Keywords:** Chemicals, Named Entity Recognition, Conditional Random Fields

## Abstract

**Background:**

The recognition of drugs and chemical entities in text is a very important task within the field of biomedical information extraction, given the rapid growth in the amount of published texts (scientific papers, patents, patient records) and the relevance of these and other related concepts. If done effectively, this could allow exploiting such textual resources to automatically extract or infer relevant information, such as drug profiles, relations and similarities between drugs, or associations between drugs and potential drug targets. The objective of this work was to develop and validate a document processing and information extraction pipeline for the identification of chemical entity mentions in text.

**Results:**

We used the BioCreative IV CHEMDNER task data to train and evaluate a machine-learning based entity recognition system. Using a combination of two conditional random field models, a selected set of features, and a post-processing stage, we achieved F-measure results of 87.48% in the chemical entity mention recognition task and 87.75% in the chemical document indexing task.

**Conclusions:**

We present a machine learning-based solution for automatic recognition of chemical and drug names in scientific documents. The proposed approach applies a rich feature set, including linguistic, orthographic, morphological, dictionary matching and local context features. Post-processing modules are also integrated, performing parentheses correction, abbreviation resolution and filtering erroneous mentions using an exclusion list derived from the training data. The developed methods were implemented as a document annotation tool and web service, freely available at http://bioinformatics.ua.pt/becas-chemicals/.

## Background

Named entity recognition (NER) refers to the task of identifying mentions of entities of specific types in natural language texts. This crucial initial task is required in order to perform other information extraction steps, such as identifying the exact concepts associated with the entity names mentioned in the texts (a task called entity normalization), identifying relations and events involving those concepts, or inferring concept characteristics and other associations [1]. One of the fields where NER has received increasing interest is biomedicine, due to its social and scientific importance and to the challenges brought by the intrinsic complexity of the domain, but also because of the overwhelming rise in the amount of published texts, namely in the form of scientific publications. Major focus was initially placed on the identification of gene and protein names, and was greatly promoted by community efforts such as the series of BioCreative challenges in biomedical text mining [2,3]. These led to the development of various systems addressing this specific task [4], with the best performing systems achieving F-measure results above 85%. These community evaluations have also shown that, at least in the case of gene and protein entity recognition, machine-learning methods generally lead to better overall results when compared to other approaches such as using dictionaries or rules, and that combining different approaches in a single hybrid system or combining different systems may also help improve the final prediction.

More recently, other types of concepts have deserved attention, including diseases, anatomy, chemicals and drugs [5-7]. In the case of diseases and anatomical concepts, dictionary-based approaches are able to provide results in the order of 65%-70%, if exact matching of the entity names is considered, or as high as 85% if a more relaxed (overlap) matching is allowed [8]. Regarding chemical entities and drugs, different approaches based on rules, dictionaries, and machine-learning have been proposed [9]. These try to cover the different nomenclatures used to reference this type of entity, including common or brand names of drugs (e.g. aspirin), chemical formulas, abbreviations, or names following a systematic convention, such as the one defined by the International Union of Pure and Applied Chemistry (IUPAC) (e.g. 2-acetoxybenzoic acid). While common or trivial names can generally be identified using a dictionary, systematic or quasi-systematic names and formulas are better managed through rule (or pattern) based approaches or by using machine-learning methods [9].

Hettne et al. [10] described the creation of a large dictionary of chemical and drug names, obtained by compiling information from seven different sources. The combined dictionary was cleaned by manual checking of highly frequent terms and by using rules for filtering and disambiguation. To evaluate the quality of the dictionary, the authors tested it on a corpus of 100 Medline abstracts [11], obtaining a precision of 67% for a recall of 40%. Corbett et al. [12], on the other hand, annotated a corpus of 42 full-text papers based on carefully revised guidelines, and evaluated the application of machine-learning methods for the recognition of chemical entities. They reported a maximum F-measure of 74% using a Hidden Markov Model (HMM) with simple token based features. The authors also underlined the impact of customizing the tokenization strategy for this task, namely regarding the processing of parenthesis and hyphens. The same corpus was also used to evaluate OSCAR3, a hybrid system combining dictionary-matching, machine-learning based on Maximum Entropy Markov Models (MEMMs), and regular expressions, to identify different types of chemical nomenclatures [13]. This system achieved a F-measure of 81% on this corpus and of 83% on a different corpus composed of 500 Medline abstracts. Klinger et al. [14] focused their work on systematic (IUPAC) and quasi-systematic names. They created a training corpus of 463 Medline abstracts, containing a total of 4751 entity mentions, and a test corpus with 1000 abstracts containing 165 entity annotations. The best performance was achieved with a third-order Conditional Random Field (CRF) model, with an F-measure of 86%.

Despite the existence of previous work on the identification of chemical entities from text, and of its agreed relevance, further developments have been hindered in part due to the limited number, scale, and lack of homogeneity of resources for training and evaluating systems for this task. To overcome these aspects, the BioCreative IV CHEMDNER task [15] was organized with the dual aim of assessing the effectiveness of using automatic tools for the identification of mentions of chemical compounds and drugs in scientific documents, while at the same time promoting the development of such tools. To achieve these objectives, a newly annotated corpus was created, composed of ten thousand abstracts selected from over 200 journals in different sub-fields of chemistry, and containing over 84 thousand entity mentions organized in seven different classes - systematic, trivial, abbreviation, family, identifiers, formula and multiple - plus a small number of unclassified annotations, accounting for around 0.13% of the total annotations in the corpus.

## Results and discussion

In this work, we propose an annotation system, based on Conditional Random Field (CRF) models, for the identification of chemical and drug names in scientific texts. This section presents the performance evaluation results obtained on the test portion of the BioCreative IV CHEMDNER task data, for the two sub-tasks proposed in this community evaluation challenge:

• Chemical Entity Mention recognition (CEM): for a given document, the aim is to provide the start and end indices for all occurrences of chemical entity names, ranked according to the confidence that the given chunk represents in fact a mention of a chemical entity;

• Chemical Document Indexing (CDI): for a given document, the task is to provide a confidence ranked list of individual chemical entities mentioned in the text.

In order to fully evaluate the performance of the proposed entity recognition system, we performed different analyses of the annotation results. Specifically, we studied the importance of specific feature types and their impact on the recall results for different entity classes, evaluated the application of a blacklist-based post-processing module, and analysed the final results in terms of exact and relaxed matching.

Furthermore, we present a web-based document annotation interface and webservices for the tagging of chemical entities in MEDLINE abstracts or in user provided texts.

### Contribution of different features

Table 1 shows the results of the feature evaluation, using first-order CRF models created with the training set documents and tested on the development set. We started by comparing the use of conjunctions against the use of windows of features. The results show that using conjunctions produces significantly better results, with an improvement of 7% in recall and of almost 3% in precision.

**Table 1:** Iterative feature elimination results.

		Precision (%)	Recall (%)	F-measure (%)
Windows		78.65	78.75	78.70
Conjunctions		81.37	85.83	83.54

Base	Token	81.32	85.86	83.53

	Lemma	78.51	83.80	81.07
Linguistic	POS	81.75	73.21	77.25
	Chunk	82.93	80.83	81.86
	Dependency parsing	85.88	82.78	**84.30**

	Capitalization	85.97	83.04	***84.48***
Orthographic	Counting	85.86	83.09	84.45
	Symbols	85.99	82.96	84.45

	Char n-grams	85.88	82.53	84.17
Morphological	Suffix	85.74	83.02	84.36
	Prefix	85.93	83.03	84.45
	Word shape	85.83	82.42	84.09

Lexicons	Chemicals	85.33	81.48	83.36

The first line shows the results obtained with the full set of features, together with windows or conjunctions of features. The following lines show results after iterative and cumulative removal of features. Values in bold indicate improvements over the previous best result; the italic value indicates the best result, obtained by removing dependency parsing and capitalization features.

Next, we evaluated the impact of each remaining feature. The following lines in Table 1 show the performance achieved when removing each of the features from our initial feature set. Note that the results shown are for the cumulative removal of features, that is, at each step we removed the feature that had the largest positive impact and on the next step this feature was no longer considered. As can be seen from the table, removing the token feature had almost no impact on the performance of the trained models. Nevertheless, given the slight negative impact on precision and F-measure, we kept this feature. Removing three of the linguistic features, namely lemmas, part-of-speech and chunk tags produced important negative impacts on F-measure. Removing the lemma had a significant impact on precision as well as recall, resulting in 2.5 percentage points decrease in F-measure. In our experiments, removing POS tags led to the largest negative impact on the results, indicating the importance of this feature for training models to recognize chemical entities. This was mostly due to the impact on recall, with a decrease of 12.6 percentage points compared to the model trained with the complete feature set. Removing chunk tags also contributed negatively to recall, with a drop of 5 percentage points, although precision improved by over 1.5%.

Noticeably, the model trained without the dependency parsing features achieved better results, in terms of F-measure, than when these features were included. This is in some way an unexpected result since these features, although computationally expensive, have been shown to contribute to better recognition performance (e.g. for genes and proteins) [19]. As can be seen, removing this feature considerably improved precision by 4.5%, while recall was lowered by 3%.

Further improvements were obtained by removing the capitalization features, in addition to removing the dependency parsing features, leading to an increase of 0.26% in recall and a slight increase in precision. Removing any of the remaining features did not produce improved results. Counting, symbols, and prefix features had almost no impact on the results when removed. Suffixes, on the other hand, showed some impact on precision which may indicate their importance for differentiating some types of chemical entity mentions from other words in the text. Conversely, character n-grams and word shape features are relevant in terms of recall, as indicated by the results.

Another interesting result is the impact on both precision and recall of removing the domain knowledge feature derived from dictionary matching. This shows the importance of this type of feature in training the machine-learning model and indicates that improving the quality of the dictionary could have an even greater impact on the final results.

### Class recall

To further understand how each feature affects the recognition results, we evaluated their impact in terms of the different chemical entity classes annotated in the corpus. Since these classes have distinct lexical characteristics, we expected that removing different feature types would have different impact on each class. However, we are not able to evaluate class-based precision, as we do not assign specific classes to the entities we recognize, and have therefore restricted to evaluating recall for each different class. Table 2 shows the results obtained on the development set, with the same models used for the overall feature evaluation shown above. The 'Unclassified' entity class was not considered, as this accounts only for 32 annotations on the development set.

**Table 2:** Impact of features on recall for each different class.

		Class recall (%)						
Feature		Multiple	Family	Abbrev	System	Formula	Identifier	Trivial
Conjunctions (all features)		42.02	82.22	81.09	90.21	79.60	73.08	91.46

Base	Token	+1.06	-0.19	+0.55	-0.09	-0.15	0.00	+0.07

	Lemma	+5.32	-2.25	-1.46	-2.95	-1.16	-4.85	-1.87
Linguistic	POS	-18.62	-12.65	-10.93	-13.88	-16.65	-24.88	-9.61
	Chunk	-22.87	-6.68	-4.34	-4.91	-6.14	-12.83	-3.13
	Dependency	0.00	-3.72	-3.76	-1.44	-4.45	-9.39	-2.52

	Capitalization	-0.53	+0.43	+0.15	+0.26	+0.31	-0.94	+0.30
Orthographic	Counting	0.00	+0.24	-0.27	+0.16	-0.12	-1.72	+0.26
	Symbols	-1.06	-0.12	-0.11	0.00	-0.24	-0.16	+0.01

	Char n-grams	+1.60	-2.51	-0.64	-0.43	-1.35	+4.07	+0.47
Morphological	Suffix	+1.06	-0.09	-0.33	+0.16	+0.51	+0.63	-0.27
	Prefix	0.00	-0.64	-0.09	+0.10	-0.05	+0.31	+0.22
	Word shape	+1.60	-0.07	-0.97	-0.18	-1.47	-2.50	-0.57

Lexicons	Chemicals	+2.66	-0.19	-2.10	-0.78	-0.75	-10.95	-2.34

Values shown are differences in percentage points to the baseline (first line).

As expected from the feature evaluation results, the POS feature is the one with most impact on the results, largely affecting the recall for all entity classes. Another feature that showed a great impact on results were chunk tags. Removing this feature affected especially the 'Multiple' and 'Identifier' classes, which are also the ones most affected by the removal of POS tags. In the case of the 'Multiple' class, this may result from the difficulty in modelling these entity mentions using the remaining features, as well as from the relatively small number of occurrences in the training set. Furthermore, removing any of the linguistic features led to significant negative impact on the recall for all the entity classes, except for lemma and dependency parsing features on the 'Multiple' class. In the case of dependency parsing features, the overall impact on recall was compensated by improvement in precision, as shown before. Capitalization features, the other type of features that contributed positively to the overall F-measure when removed, shows different results when analyzing the impact on each class, with reductions in recall for 'Multiple' and 'Identifier', and small improvements for the remaining classes.

As can be seen, removing counting and word shape features, although with no significant impact on the overall results, led to reductions on the recall for the 'Identifier' class. On the other hand, character n-gram features seem to be unnecessary for recognizing entities of this class. Removing domain knowledge features had a significant impact on the recall for 'Trivial' and 'Abbreviation' entity classes, as expected, but proved even more relevant for the 'Identifier' class. This seems to indicate that, because different types of identifiers exist, their recognition is largely dependent on being present in the dictionary, as well as on their linguistic role on the sentence, as denoted by the importance of the linguistic features for this entity class.

### Error analysis

In order to identify the most common annotation errors produced by the model, we performed an error analysis of the results. As before, we based this analysis on the results obtained on the development set, with models trained on the training part of the corpus.

A simple analysis showed, as expected, that a large number of false-positive annotations is produced by shorter annotations. In fact, 18.1% of the false-positives stem from annotations with one or two characters in length. In comparison, these short mentions correspond to 5.3% of the gold-standard annotations in the development part of the corpus. If annotations up to three characters in length are considered, then these correspond to 31.3% of the total false-positives produced, but account only for 15.8% of the gold-standard annotations. However, applying a blind filtering based on annotation length alone degrades the evaluation results. In the development set, removing annotations with a single character improves precision by only 0.2% while degrading recall by 0.8%. If a more stringent filter is applied, by removing annotations with three characters or less, recall drops by 12.6 percentage points while precision only improves slightly, by 2.6%.

We therefore tried to define an exclusion list for filtering the annotations produced by the recognition model, by first calculating the log odds ration between the number of true positive counts and the number of false positive counts for each distinct (case-insensitive) mention predicted by the model. We calculated this in ten folds and, in each fold, we varied the filtering threshold used to remove annotations from the 10% of documents used for testing. Additionally, annotations were only added to this exclusion list if they resulted in a minimum number of false positives in the 90% of the corpus used in each fold to define these thresholds. This number was also varied to identify the best value. Using this strategy, we observed that an average improvement of 0.6% in F-measure could be achieved with an exclusion list containing the annotations with a log odds ratio between true-positive and false-positive counts lower than 0.3, and appearing at least two times as false-positives. Applying these thresholds to the entire development set, an exclusion list containing 218 annotations was produced.

### Final evaluation results

Based on the results of the feature evaluation, we used the best feature set (i.e. removing dependency parsing and capitalization features) to train various CRF models of orders 1 to 4. Applied separately, higher-order models produced similar or slightly inferior results when compared to first-order models (between -0.02% and -0.48% reduction in F-measure). Given that training times increase exponentially with the model order, we removed third and fourth-order models from further analysis. Therefore, we trained first and second-order CRF models on the combined training and development sets of the CHEMDNER corpus (a total of 7000 abstracts) and used these to annotate the test set.

The results of annotating the documents in the test set using the harmonized predictions from the two CRF models are shown in Table 3. The first lines in the table show the official results of the best performing runs from all teams participating in each sub-task of the CHEMDNER challenge, and of our best submitted run. The following line shows the results of the same submitted run, after correcting minor errors in the generation of the annotation files, identified after the challenge. Also shown in the table are the results obtained with the first- and second-order CRF models, when used separately.

**Table 3:** Final evaluation results on the CHEMDNER test set.

	Entity Mention			Document Indexing		
System	Precision	Recall	F-measure	Precision	Recall	F-measure
Top scoring	89.09	85.75	87.39	87.02	89.41	88.20

Official best run	86.50	85.66	86.08	86.35	82.37	84.31
Corrected run	87.35	86.49	86.92	87.07	87.97	87.52
	+0.85	+0.83	+0.84	+0.72	+5.60	+3.21
1st order CRF	88.04	84.89	86.44	88.00	86.42	87.20
	+0.69	-1.60	-0.48	+0.93	-1.55	-0.31
2nd order CRF	88.35	83.79	86.01	88.14	86.65	87.39
	+1.01	-2.71	-0.91	+1.08	-1.32	-0.13
Post-processing	88.67	86.32	87.48	87.68	87.81	87.75
	+1.32	-0.17	+0.56	+0.61	-0.16	+0.23

The 'Corrected run' line shows results obtained using the same models as in the official run, after correcting the generation of the annotation files. Results obtained with first- and second-order CRF models alone (without model combination) and after post-processing are shown with differences compared to the corrected run. Values are shown in percentage points.

Considering exact matching between the predictions and gold standard annotations, our system achieves an F-measure of 86.92%, with a precision of 87.35% and a recall of 86.49% in the test set of the chemical entity mention task. Comparing to the use of the first-order CRF model, the harmonized predictions show an improvement of 0.48% in F-measure. As can be seen, although the second-order model leads to slightly worst results, using in combination with the first-order model helps improve recall by 1.6%, significantly contributing to the final performance results.

Applying the exclusion list defined above, the performance improves slightly, achieving an F-measure of 87.48% (+0.56%), with only a small drop in recall (-0.17%). These results compare favourably with the best results obtained during the BioCreative CHEMDNER task, where the best system obtained an F-measure of 87.39%, for a precision of 89.09% and a recall of 85.75%.

Regarding the chemical document indexing subtask of the BioCreative CHEMDNER evaluation challenge, our system achieves an F-measure of 87.52%, for a precision of 87.07% and a recall of 87.97%. The application of the exclusion list had a lower impact on this task, improving F-measure to 87.75% (+0.23%) with a decrease of 0.16% in recall. The model combination also had lesser impact on the results of this task, which could be justified by the fact that only one of the occurrences of each distinct entity in a text needs to be correctly identified.

**Figure 1:**
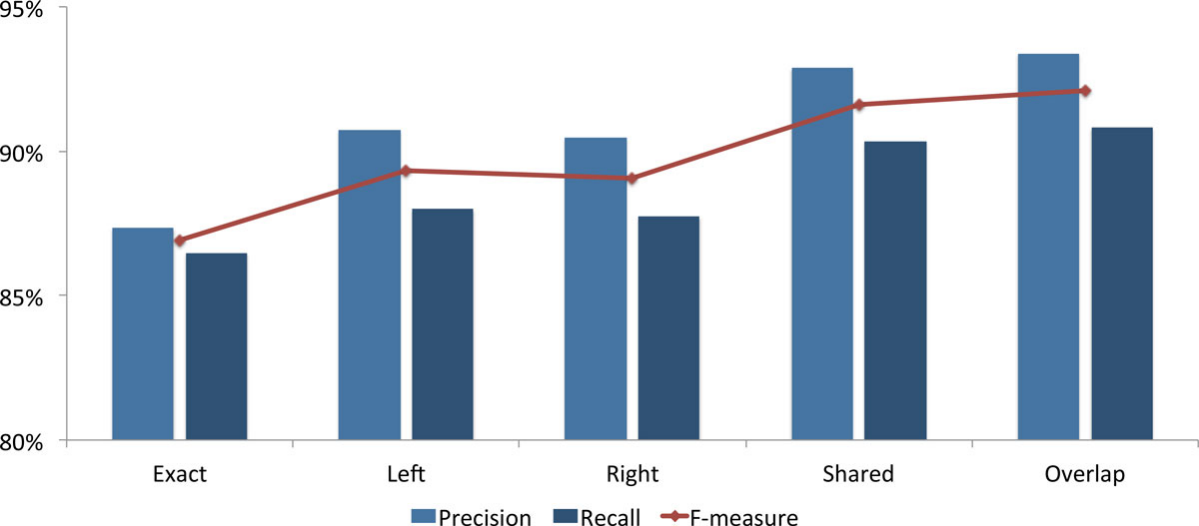
**Results obtained on the CHEMDNER test set, using a combination of a first-order and a second-order CRF model, trained using the selected feature set**.

Figure 1 illustrates the entity mention recognition results in more detail, by comparing exact matching to other less stringent matching strategies: left matching, meaning that the start indices of the predicted mention and the gold-standard annotation match; right matching, when the end indices match; shared, when either start or end indices match; and overlap, which considers any kind of intersection between the predicted and gold standard mentions. Analysing the results of these different matching methods allows obtaining a better understanding of the types of errors generated, and may provide hints on how to overcome some of those errors. Additionally, some following information extraction and document annotation steps may still be performed correctly, even with small errors in the matching of mentions. For example, considering left and right matching, the precision of the results go up by 3.38 and 3.11 percentage points, and recall also improves by 1.51 and 1.25 points, respectively. This corresponds to 787 (721 for right matching) annotations being correct if the start (end) index of the mention is considered. If either matching is allowed, then F-measure increases to 91.60%, with a precision of 92.89% and a recall of 90.34%. These results give a better indication of the system performance and effectiveness for tasks such as text-mining assisted bio-curation.

### Annotation service and web interface

**Figure 2:**
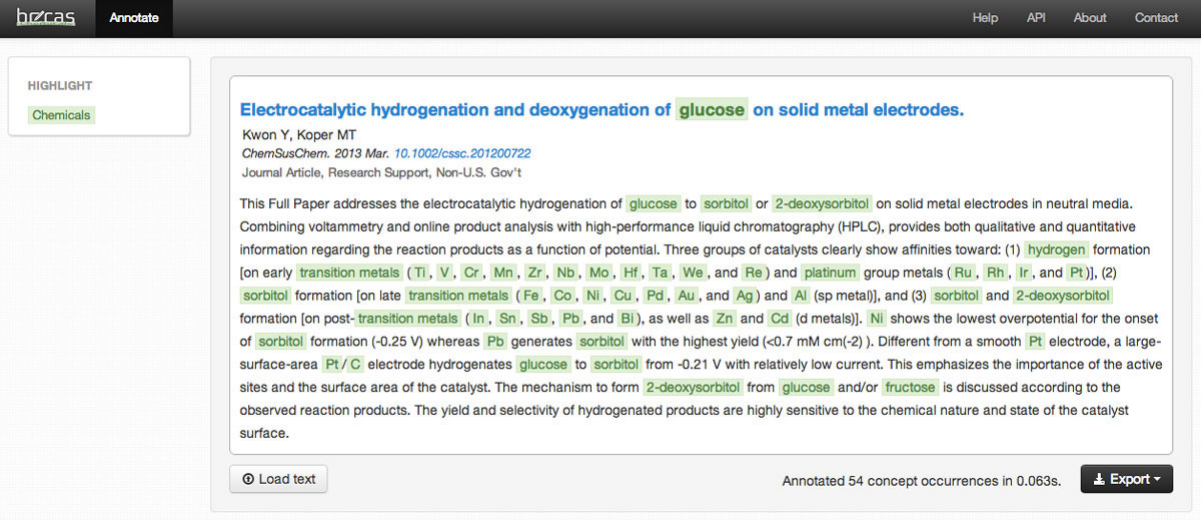
**Web interface for recognition and annotation of chemical entities in text**. Available at: http://bioinformatics.ua.pt/becas-chemicals

The chemical entity recognition system described in this work can be used through a web-based application and a REST API for document annotation, available at http://bioinformatics.ua.pt/becas-chemicals/. These services are based on the more general Becas service, a web application and API for customizable annotation of concepts from 11 distinct semantic groups [16]. Figure 2 shows the resulting annotation for a MEDLINE abstract, with 54 concept mentions identified in the title and abstract. Free text can also be uploaded or copied into the annotation window for processing.

## Conclusion

This article presents a CRF-based solution for automatic chemical and drug name recognition. The implemented pipeline takes advantage of a rich feature set, namely linguistic, orthographic, morphological, domain knowledge (through dictionary matching) and local context (through conjunctions) features. Post-processing modules are also integrated, performing parentheses correction and abbreviation resolution. In the end, a first- and second-order CRF models are harmonized to obtain improved annotations. The final performance results achieved in the BioCreative IV CHEMDNER test set, with F-measures of 87.48% for chemical entity mention and 87.75% for chemical document indexing, demonstrate the effectiveness of the method. The additional filtering step, using an exclusion list derived from the development set, resulted in improved performance in the CEM task, with an increase of 1.32% in precision and 0.56% in F-measure.

The entity recognition system described in this work was developed on top of two frameworks providing efficient methods for document processing, feature extraction, training machine learning (ML) models, and for multi-threaded document annotation. The tool is freely available through an end-user web tool and as a document annotation API.

## Methods

We applied a supervised machine-learning approach, through the application of Conditional Random Fields (CRFs) [17] provided by MALLET [18]. Additionally, we compiled a dictionary of chemical entity name, and used the matches of these names in the texts as features for the CRF model.

**Figure 3:**
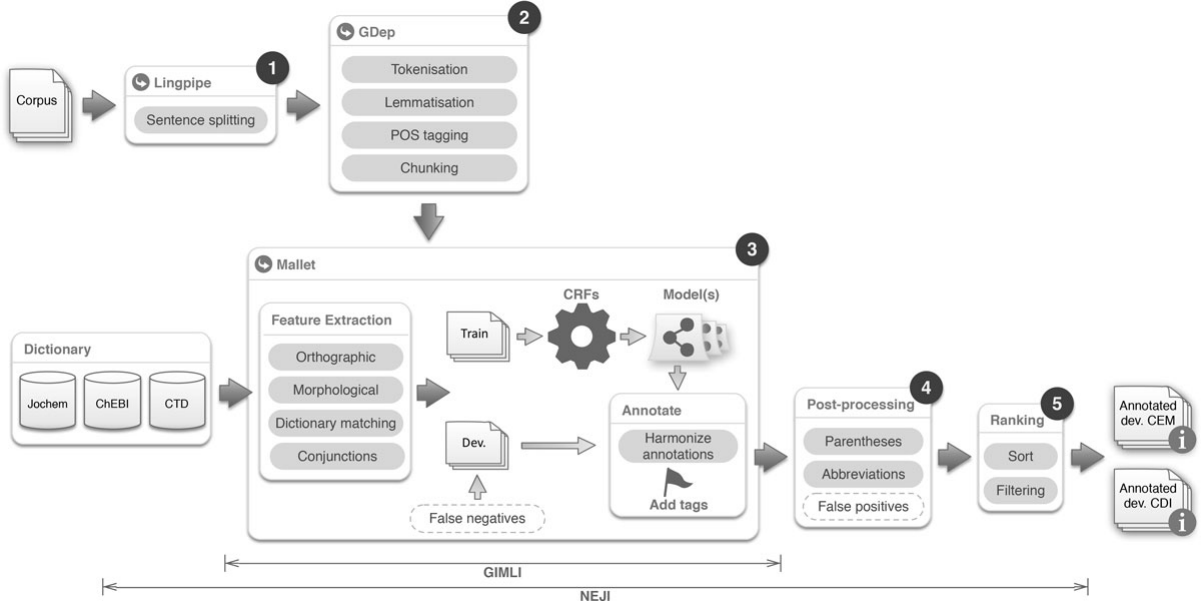
**Overall architecture of the described solution, presenting the pipeline of required steps, tools and external resources**. Boxes with dotted lines indicate optional processing modules.

The method applied for this work was developed on top of two frameworks: Gimli [19] was used for feature extraction and to train the machine learning (ML) models, and Neji [8] was used for pre- and post-processing tasks and as the framework for multi-threaded document annotation. Figure 3 illustrates the overall architecture and the steps performed.

### Corpus and evaluation metrics

The system described in this work was trained and evaluated on the BioCreative IV CHEMDNER corpus [15], which is provided in three sub-sets: a training set containing 3500 Medline abstracts annotated with 29478 mentions of chemical entities, a development set composed of 3500 abstracts with 29526 entity mentions, and a test set composed of 3000 abstracts, and containing 25351 mentions. Seven chemical entity classes were defined in the corpus annotation guidelines. However, instead of treating each class separately, we grouped all classes into a single class.

The training and development sets were used to train and refine the machine learning models, and to perform the feature evaluation studies. The final model was trained on the combined training and development set and evaluated on the test set.

The common evaluation metrics were used, namely *Recall *= *TP*/(*TP *+ *FN*), *Precision *= *TP/*(*TP *+ *FP*) and *F*1 = 2 × *Precision *× *Recall*/(*Precision *+ *Recall*), were TP refers to true positives, FP to false positives, and FN refers to false negatives.

### Corpus pre-processing

As shown in Figure 3, the first fundamental step is to perform sentence splitting, in order to divide the texts in the basic units of logical thought. For performing this step, we take advantage of Lingpipe [20], which provides a model trained on biomedical corpora that presents high-performance results [21]. The following Natural Language Processing (NLP) tasks are achieved through a customized version of GDep [22], a dependency parser for the biomedical domain built on top of the GENIA tagger that performs tokenization, lemmatization, part-of-speech (POS) tagging and chunking. We modified the tokenizer in GDep so that words containing the symbols "/", "-" or "." are always divided into multiple tokens, making its behaviour more consistent. This simple change proved to be effective when applied to gene/protein entity recognition in different corpora [19]. Finally, the corpus annotations were encoded with the BIO scheme.

### Feature set

Given the rich and heterogeneous characteristics of chemical names, we defined a complex feature set to properly represent these entities, including token and NLP derived features, orthographic and morphologic characteristics, domain knowledge, provided by the occurrence of known terms from a purpose-built dictionary, and local context information:

• NLP features:

- Token, lemma, POS and chunk tags;

- Dependency parsing

• Orthographic features:

- Capitalization (e.g., "StartCap" and "AllCaps");

- Digits and capitalized characters counting (e.g., "TwoDigit" and "TwoCap");

- Symbols (e.g., "Dash", "Dot" and "Comma");

- Greek letters (e.g., features for "alpha" and "*α*").

• Morphological features:

- Suffixes, prefixes and char n-grams of 2, 3 and 4 characters;

- Word shape features to reflect how letters, digits and symbols are organized in the token (e.g., the structure of "Abc:1234" is expressed as "Aaa#1111").

• Domain knowledge:

- Dictionary matching using a combined dictionary with terms from Jochem [10], ChEBI [23] and CTD [24].

• Local context:

- Lemma, POS and chunk features, extracted from the windows {-1, 0}, {-2, -1}, {0, 1}, {-1, 1} and {-3, -1} around the current token;

- Conjunctions of lemma and POS features, from the same windows.

To encode the domain knowledge features, we performed preliminary tests on the training and development data, using a set of dictionaries compiled from different sources including also PubChem [25] and Drugbank [26]. Based on these initial tests, the dictionaries extracted from Jochem, ChEBI and CTD were selected, as their combination led to the best results when used in a dictionary-matching approach as well as when used to train a machine-learning model with a reduced set of features.

In order to select the best feature set for this entity recognition task, we followed an iterative elimination approach, starting with the full feature set defined above and using the development data to evaluate models trained on the training data. We started by comparing the two methods of encoding local context information, using windows or conjunctions as defined above, together with the other features. When using windows, selected features from preceding and succeeding tokens are directly added as features for the current token. In the case of conjunctions, on the other hand, new features are created, consisting on the concatenation of selected features from the surrounding tokens. In this work, we used lemmas, POS and chunk tags from neighbouring tokens to encode local context through windows, and the concatenation of lemmas and POS tags to encode local context through conjunctions. These features were selected after preliminary tests performed on the training data. Having selected the preferred local context feature, we evaluated the importance of the remaining feature types by iteratively removing each one and checking the impact on the model's performance. Whenever removing a given feature led to improved results, it was eliminated from the set and was therefore not considered in the next iteration. This process was repeated until no other feature could be removed without negatively affecting the results.

### Machine-learning models

Since most recent results on biomedical NER indicate that better performance results can be obtained by combining annotations from systems with different characteristics [27], we initially considered CRF models with orders 1 to 4 to achieve such heterogeneity. Additionally, we tested CRF models with forward (from left to right) and backward (from right to left) parsing. However, contrary to the positive contribution in the case of gene and protein entities, backward parsing models did not provide positive outcomes in this task, when tested on the development data.

A simple algorithm was used to harmonize annotations provided by CRF models with different orders. Basically, all the annotations with non-overlapping spans are added to the final list of annotations. In the case two (or more) annotations from different CRF models intersect, the one with the highest confidence score as calculated by the model is selected, while the remaining ones are ignored.

### Post-processing steps

In order to solve some errors generated by the CRF model, our solution integrates two mandatory post-processing modules, implementing parentheses correction and abbreviation resolution. To perform parentheses correction, the number of parentheses (round, square and curly) on each annotation is verified and the annotation is removed if this is an odd number, since it clearly indicates a mistake by the ML model. Regarding abbreviation resolution, we adapted a simple but effective abbreviation definition recognizer [28], which is based on a set of pattern-matching rules to identify abbreviations and their full forms. Thus, if one of the forms is annotated as an entity name, the other one is added as a new annotation. Additionally, if one of the forms is not completely annotated, we expand the annotation boundaries using the result from the abbreviation extraction tool.

Additionally, we performed an error analysis on the results achieved on the development set using a CRF model trained on the training set, in order to collect false positive and false negative annotations. We compared the number of times a given chunk of text was correctly annotated by the system (a true positive) to the number of times it was incorrectly annotated (a false positive), and calculated the log odds ratio of these counts. These odds ratios could then be used to select an exclusion list for improving the precision of the results. Furthermore, in order to determine which terms to use in this exclusion list, we defined two thresholds, one for the log odds value and the second one for the minimum times a chunk was found as a false positive. To select the optimum value for these thresholds, we performed a 10-fold analysis on the development set.

### Ranking the annotations

The final step of the annotation pipeline is to rank the predicted annotations, so that annotations that are most likely correct appear at the top. Ranking is derived from the confidence scores provided by the CRF models, a value between 0 and 1 that reflects the certainty of the model generating each annotation. In that way, ranking simply orders the annotations in descending order of scores. In the case of the CDI task, an additional filtering step is applied to remove repeated matches of the same terms in each text.

## Competing interests

The authors declare that they have no competing interests.

## Authors' contributions

DC conceived, designed and ran the experiments and participated in results analysis. SM analyzed the data and results and wrote the initial manuscript. JLO participated in study design and data analysis. All authors reviewed and approved the final manuscript.

## Declarations

Publication funding for this article has come from FCT - Foundation for Science and Technology, in the context of the project PEst-OE/EEI/UI0127/2014.

This article has been published as part of *Journal of Cheminformatics *Volume 7 Supplement 1, 2015: Text mining for chemistry and the CHEMDNER track. The full contents of the supplement are available online at http://www.jcheminf.com/supplements/7/S1.

## References

[B1] Campos D Matos S Oliveira JL Current Methodologies for Biomedical Named Entity Recognition 2013 John Wiley & Sons, Inc., Hoboken, New Jersey 839 868

[B2] Smith L Tanabe LK Ando RJn Kuo C-J Chung I-F Hsu C-N Lin Y-S Klinger R Friedrich CM Ganchev K Torii M Liu H Haddow B Struble CA Povinelli RJ Vlachos A Baumgartner Wa Hunter L Carpenter B Tsai RT-H Dai H-J Liu F Chen Y Sun C Katrenko S Adriaans P Blaschke C Torres R Neves M Nakov P Divoli A Manã-lópez M Mata J Wilbur WJ Overview of BioCreative II gene mention recognition Genome Biology 2008 9 Suppl 2 2 10.1186/gb-2008-9-s2-s2 PMC2559986 18834493

[B3] Lu Z Kao H-Y Wei C-H Huang M Liu J Kuo C-J Hsu C-N Tsai RT-H Dai H-J Okazaki N Cho H-C Gerner M Solt I Agarwal S Liu F Vishnyakova D Ruch P Romacker M Rinaldi F Bhattacharya S Srinivasan P Liu H Torii M Matos S Campos D Verspoor K Livingston KM Wilbur WJ The gene normalization task in BioCreative III BMC bioinformatics 2011 12 Suppl 8 2 10.1186/1471-2105-12-S8-S2 22151901 PMC3269937

[B4] Campos D Matos S Oliveira JL Sakurai S Biomedical Named Entity Recognition: A Survey of Machine-Learning Tools Theory and Applications for Advanced Text Mining, InTech, Rijeka, Croatia 2012 175 195

[B5] Ohta T Pyysalo S Tsujii J Ananiadou S Open-domain anatomical entity mention detection Proceedings of the Workshop on Detecting Structure in Scholarly Discourse. ACL '12, Association for Computational Linguistics, Stroudsburg, PA, USA 2012 27 36

[B6] Doǧan RI Lu Z An improved corpus of disease mentions in PubMed citations Proceedings of BioNLP'12. Association for Computational Linguistics, Stroudsburg, PA, USA 2012

[B7] Bada M Eckert M Evans D Garcia K Shipley K Sitnikov D Baumgartner Wa Cohen KB Verspoor K Blake JA Hunter LE Concept annotation in the CRAFT corpus BMC bioinformatics 2012 13 161 10.1186/1471-2105-13-161 22776079 PMC3476437

[B8] Campos D Matos S Oliveira JL A modular framework for biomedical concept recognition BMC bioinformatics 2013 14 281 24063607 10.1186/1471-2105-14-281 PMC3849280

[B9] Vazquez M Krallinger M Leitner F Text Mining for Drugs and Chemical Compounds: Methods, Tools and Applications Molecular Informatics 2011 30 6-7 506 519 10.1002/minf.201100005 27467152

[B10] Hettne KM Stierum RH Schuemie MJ Hendriksen PJM Schijvenaars BJa Mulligen EMv Kleinjans J Kors Ja A dictionary to identify small molecules and drugs in free text Bioinformatics (Oxford, England) 2009 25 22 2983 2991 10.1093/bioinformatics/btp535 19759196

[B11] Kolárik C Klinger R Friedrich CM Hofmann-Apitius M Fluck J Chemical names: terminological resources and corpora annotation Workshop on Building and Evaluating Resources for Biomedical Text Mining (Language Resources and Evaluation Conference) 2008 51 58

[B12] Corbett P Batchelor C Teufel S Annotation of chemical named entities Proceedings of the Workshop on BioNLP 2007: Biological, Translational, and Clinical Language Processing. BioNLP '07. Association for Computational Linguistics, Stroudsburg, PA, USA 2007 57 64

[B13] Corbett P Copestake AA Cascaded classifiers for confidence-based chemical named entity recognition BMC Bioinformatics 2008 9 S-11 19025690 10.1186/1471-2105-9-S11-S4 PMC2586753

[B14] Klinger R Kolárik C Fluck J Hofmann-Apitius M Friedrich CM Detection of IUPAC and IUPAC-like chemical names Bioinformatics (Oxford, England) 2008 24 13 268 76 10.1093/bioinformatics/btn181 PMC2718657 18586724

[B15] Krallinger M Leitner F Rabal O Vazquez M Oyarzabal J Valencia A Overview of the chemical compound and drug name recognition (chemdner) task BioCreative Challenge Evaluation Workshop 2013 2 2

[B16] Nunes T Campos D Matos S Oliveira JL BeCAS: biomedical concept recognition services and visualization Bioinformatics (Oxford, England) 2013 29 15 1915 1916 10.1093/bioinformatics/btt317 23736528

[B17] Lafferty J McCallum A Pereira F Conditional random fields: Probabilistic models for segmenting and labeling sequence data 2001

[B18] McCallum AK MALLET: A Machine Learning for Language Toolkit http://mallet.cs.umass.edu

[B19] Campos D Matos S Oliveira JL Gimli: open source and high-performance biomedical name recognition BMC bioinformatics 2013 14 1 54 10.1186/1471-2105-14-54 23413997 PMC3651325

[B20] Alias-i I LingPipe http://alias-i.com/lingpipe/index.html

[B21] Verspoor K Cohen KB Lanfranchi A Warner C Johnson HL Roeder C Choi JD Funk C Malenkiy Y Eckert M Xue N Baumgartner Wa Bada M Palmer M Hunter LE A corpus of full-text journal articles is a robust evaluation tool for revealing differences in performance of biomedical natural language processing tools BMC bioinformatics 2012 13 207 10.1186/1471-2105-13-207 22901054 PMC3483229

[B22] Sagae K Dependency parsing and domain adaptation with LR models and parser ensembles Eleventh Conference on Computational Natural Language Learning, Prague, Czech Republic. Association for Computational Linguistics 2007 1044 1050

[B23] Degtyarenko K De Matos P Ennis M Hastings J Zbinden M McNaught A Alcántara R Darsow M Guedj M Ashburner M ChEBI: a database and ontology for chemical entities of biological interest Nucleic acids research 2008 36 suppl 1 344 350 17932057 10.1093/nar/gkm791 PMC2238832

[B24] Davis AP Murphy CG Saraceni-Richards CA Rosenstein MC Wiegers TC Mattingly CJ Comparative Toxicogenomics Database: a knowledgebase and discovery tool for chemical-gene-disease networks Nucleic acids research 2009 37 Database 786 92 10.1093/nar/gkn580 PMC2686584 18782832

[B25] Wang Y Xiao J Suzek TO Zhang J Wang J Bryant SH Pubchem: a public information system for analyzing bioactivities of small molecules Nucleic acids research 2009 37 suppl 2 623 633 19498078 10.1093/nar/gkp456 PMC2703903

[B26] Wishart DS Knox C Guo AC Shrivastava S Hassanali M Stothard P Chang Z Woolsey J DrugBank: a comprehensive resource for in silico drug discovery and exploration Nucleic acids research 2006 34 Database 668 72 16381955 10.1093/nar/gkj067 PMC1347430

[B27] Campos D Matos S Lewin I Oliveira JL Rebholz-Schuhmann D Harmonization of gene/protein annotations: towards a gold standard MEDLINE Bioinformatics (Oxford, England) 2012 28 9 1253 1261 10.1093/bioinformatics/bts125 22419783

[B28] Schwartz AS Hearst MA A simple algorithm for identifying abbreviation definitions in biomedical text Pacific Symposium on Biocomputing, Hawaii, HI, USA 2003 Computer Science Division, University of California, Berkeley, Berkeley, CA 94720, USA 451 462 12603049

